# Obituary

**Published:** 1883-03

**Authors:** 


### Obituary.

Died Sabbath morning, March 4th, at 2 o'clock A. M., in Louisville, Ky., of chronic asthma, Dr. W. H. Goddard, in the seventy-fifth year of his age.

This announcement, though not a great surprise to those who were fully aware of the condition of the Doctor's health for the past few months, will bring sorrow to *all* who knew him, and most to those who knew him best.

To the members of the American Dental Association will this removal come with especial sadness. Dr. G. had for fifteen years served this body in one of its leading and most important offices, and with an efficiency and faithfulness not to be excelled, if equaled.

He was at the last meeting of the body elected its presiding officer, an honor which would long ago have been conferred upon him, had the Association felt that he could be spared from the position which he was so well filling.

The April, or, at furthermost, May number of the REGISTER will contain a biographical sketch of the Doctor which will, we doubt not, be of great interest to a large number in the profession.

BOARD OF DENTAL EXAMINERS OF ALABAMA.

The State Board of Dental Examiners of Alabama will hold their third annual meeting in Montgomery, Alabama, commencing the second Tuesday, the 10th day of April, 1883, at the same time and place, with the Alabama Dental Association. All parties desiring to practice dentistry in Alabama must make application for license on the first day of session.

T. M. ALLEN, D.D.S.
Eufaula, Alabama. Secretary B. of D. E., of Alabama.

